# Complement System Inhibition Modulates the Inflammation Induced by the Venom of *Premolis semirufa*, an Amazon Rainforest Moth Caterpillar

**DOI:** 10.3390/ijms232113333

**Published:** 2022-11-01

**Authors:** Joel J. M. Gabrili, Isadora Maria Villas-Boas, Giselle Pidde, Carla Cristina Squaiella-Baptistão, Trent M. Woodruff, Denise V. Tambourgi

**Affiliations:** 1 Immunochemistry Laboratory, Instituto Butantan, São Paulo 05503-900, Brazil; 2 School of Biomedical Sciences, University of Queensland, Brisbane, QLD 4072, Australia

**Keywords:** envenomation, *Premolis semirufa*, pararamosis, inflammation, complement system

## Abstract

The caterpillar of the *Premolis semirufa* moth, commonly called Pararama, is found in the Brazilian Amazon region. Contact with the hairs can cause a chronic inflammatory reaction, termed “pararamosis”. To date, there is still no specific treatment for pararamosis. In this study, we used a whole human blood model to evaluate the involvement of the complement in the proinflammatory effects of *P. semirufa* hair extract, as well as the anti-inflammatory potential of complement inhibitors in this process. After treatment of blood samples with the *P. semirufa* hair extract, there was a significant increase in the generation of soluble terminal complement complex (sTCC) and anaphylatoxins (C3a, C4a, and C5a), as well as the production of the cytokines TNF-α and IL-17 and the chemokines IL-8, RANTES, MIG, MCP-1, and IP-10. The inhibition of C3 with compstatin significantly decreased IL-17, IL-8, RANTES, and MCP-1 production. However, the use of the C5aR1 antagonist PMX205 promoted a reduction in the production of IL-8 and RANTES. Moreover, compstatin decreased CD11b, C5aR1, and TLR2 expression induced by *P. semirufa* hair extract in granulocytes and CD11b, TLR4, and TLR2 in monocytes. When we incubated vascular endothelial cells with extract-treated human plasma, there was an increase in IL-8 and MCP-1 production, and compstatin was able to decrease the production of these chemokines. C5aR1 antagonism also decreased the production of MCP-1 in endothelial cells. Thus, these results indicate that the extract of the Pararama bristles activates the complement system and that this action contributes to the production of cytokines and chemokines, modulation of the expression of surface markers in leukocytes, and activation of endothelial cells.

## 1. Introduction

Pararamosis is an occupational disease of an inflammatory nature caused by contact with the hairs of the caterpillar or cocoon of the *Premolis semirufa* moth [1]. The term pararamosis is used in reference to the popular name given to the larval form of this moth, “Pararama”. This caterpillar is native to the Amazonian region and lives on rubber trees (*Hevea* spp), feeding on its leaves. The highest frequency of accidents occurs when rubber extractors collect latex and get in contact with caterpillar hairs containing the venom [1,2,3].

Contact with the caterpillar hairs through the skin causes immediate reactions of intense itching, followed by pain, heat, and redness, typical of an acute inflammatory process lasting 3 to 7 days [1]. However, in some injured individuals, the process can become chronic with synovial membrane thickening, which can evolve to deformities and immobilization of the affected joint, configuring the clinical picture of ankylosis, similar, in some aspects, to rheumatoid arthritis (RA) [2,3] and osteoarthritis (OA) [4,5]. There is still no specific treatment for this disease, but corticosteroids have been used to prevent the development or alleviate chronic disease [6,7].

Our group previously investigated the biological characteristics and immune response to the Pararama hair extract to identify possible targets for the treatment of this disease. We demonstrated that the hair extract of *P. semirufa* possesses a mixture of enzymes that, acting together, can promote the clinical manifestations of pararamosis. In addition, repeated inoculations of Pararama hair extract in the mouse paw induced an important local inflammatory response with neutrophilic infiltrate [8]. Continuing the evaluation of local inflammation, the presence of macrophages and neutrophils was observed, possibly due to the increased production of cytokines (TNF-α, IL-1, IL-6, IL-2, IFN-γ, IL-12, IL-17, and IL-23), as observed at the inoculation site [9].

More recently, increased production of IL-6, IL-8, MCP-1, prostaglandin E2, metalloproteinases (MMP-1, MMP-2, MMP-3, and MMP-13), and complement system components (C3, C4, and C5) was observed in chondrocytes cultured with *P. semirufa* hair extract. In addition, a significant decrease in both aggrecan and type II collagen and an increase in HMGB1 protein were shown after extract treatment. This study also identified important pathways related to the inflammatory process of joint diseases, such as the inflammatory response, chemotaxis of immune cells, and extracellular matrix (ECM) remodeling [5].

In addition to the participation of cytokines, macrophages, and neutrophils in the immune response triggered by Pararama hair extract, in vitro experiments showed that, using human serum, the extract also activates the complement system, generating anaphylatoxins (C3a, C4a, and C5a) and the soluble terminal complement complex (sTCC) [10].

The complement system is involved in the pathogenesis and clinical manifestations of several systemic autoimmune diseases, such as systemic lupus erythematosus (SLE) [11], vasculitis [12], antiglomerular basement membrane disease [13], antiphospholipid antibody syndrome [14], systemic sclerosis [15], dermatomyositis [16], and rheumatoid arthritis [17], as well as in the pathogenesis and progression of osteoarthritis [18,19]. In the last 60 years, several studies have shown that uncontrolled activation of the complement system is involved in the development and/or increase in inflammatory arthritis [20,21,22,23,24,25,26,27,28,29,30,31,32,33,34]. These data indicate that anti-complement therapies may have considerable potential as a strategy for the treatment of inflammatory arthritis.

Several compounds that interfere with the complement cascade have been studied and entered into clinical trials [35,36]. For example, the humanized monoclonal antibody against C5 (eculizumab), which inhibits its cleavage to C5a and C5b, promoted a significant improvement in the clinical score of rheumatoid arthritis when administered intravenously [37]. Other compounds directed to the complement system have already been used in animal models of inflammatory diseases, such as serine protease inhibitors (FUT-175 and BCX-1470), C3 inhibitors (compstatin, Cp40), C5 inhibitors (TS-A12/22 and K76COONa), and C3a (SB290157) and C5a (PMX-53) antagonists [35,38].

Most of the in vitro experiments carried out with the aim of investigating complement system participation in different types of cells are performed using proteins and isolated cells. However, to study the role of the complement system as a mediator of inflammation, it is necessary that all soluble or membrane mediators be present and able to interact. For this interaction to occur, the ex vivo human whole blood model is an ideal approach [39]. Thus, the objective of the present study was to evaluate the involvement of the complement system in the proinflammatory effects of *Premolis semirufa* hair extract using a human whole blood ex vivo model. We also used specific complement inhibitors of key points in the cascade to determine mechanistically the drivers of the inflammatory process in this model.

## 2. Results

### 2.1. Pararama Hair Extract Activates the Complement System in Human Blood

**Figure 1:**
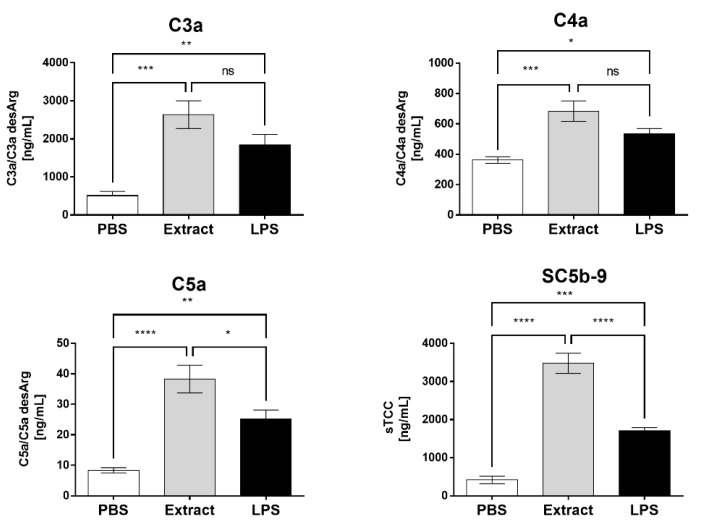
Production of anaphylatoxins and the terminal complement complex in whole blood stimulated with Pararama hair extract. Human blood samples anticoagulated with lepirudin (50 µg/mL) were incubated with Pararama hair extract (95 µg/mL), LPS (5 µg/mL), or PBS for 30 min at 37 °C. After plasma collection, anaphylatoxin (C3a, C4a, and C5a) production was assessed by flow cytometry, and SC5b-9 was assessed by ELISA. The results are presented as the mean ± SD of three independent experiments. * *p*: < 0.05. ** *p*: < 0.01. *** *p*: < 0.001. **** *p*: < 0.0001. ns: not significant.

To evaluate whether the Pararama hair extract induces complement system activation and, consequently, the generation of anaphylatoxins (C3a, C4a, and C5a) and soluble terminal complement complex (sTCC and SC5b-9), lepirudin-treated blood samples were incubated with the extract and, as a positive control, with lipopolysaccharide (LPS). Figure 1 shows that both the extract and LPS induced a significant increase in the generation of the anaphylatoxins C3a, C4a, and C5a, and the extract induced higher production of C5a than LPS. Moreover, the extract induced a significant generation of sTCC when compared to the negative control, PBS. This production was significantly higher than the production induced by LPS.

### 2.2. Pararama Hair Extract Induces Increased Expression of Cell Surface Markers in Human Blood

To assess whether the hair extract from the *P. semirufa* caterpillar had any effect on leukocytes, human blood samples treated with buffer, LPS or extract were analyzed by flow cytometry for the expression of the surface markers CD11b, CD14, TLR4, TLR2, C3aR, and C5aR1 in granulocyte (CD66b+) and monocyte (CD33+) populations, and the results are expressed as the median fluorescence intensity (MFI).

**Figure 2:**
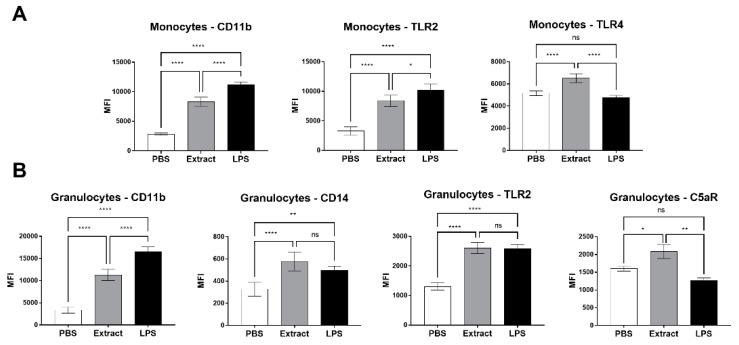
Expression of surface markers in monocytes and granulocytes stimulated with Pararama hair extract. Human blood samples anticoagulated with lepirudin (50 µg/mL) were incubated with Pararama hair extract (95 µg/mL), LPS (5 µg/mL), or PBS for 30 min at 37 °C. After lysis of erythrocytes, cells were stained with anti-CD33 (monocytes), anti-CD66b (granulocytes), anti-CD11b, anti-CD14, anti-TLR2, anti-TLR4 and anti-C5aR. The expression of these markers was evaluated by flow cytometry, and the results are expressed as the median fluorescence intensity (MFI) after the acquisition of 20,000 events. The results are presented as the mean ± SD of three independent experiments. (**A**): Monocytes. (**B**): Granulocytes. * *p*: < 0.05. ** *p*: < 0.01. **** *p*: < 0.0001. ns: not significant.

Figure 2A shows that the extract induced a significant increase in the expression of CD11b, TLR2, and TLR4 in monocytes. When compared to the Pararama hair extract, LPS induced higher expression of CD11b and TLR2. The expression of CD14, C3aR, and C5aR1 protein was not modulated by the treatments in the monocyte population (data not shown).

Figure 2B shows a significant increase in the expression of CD11b, CD14, TLR2, and C5aR1 in the granulocyte population when compared to the negative control (PBS). LPS induced a higher expression of CD11b than the extract. The expression of TLR4 and C3aR molecules was not modulated by the treatments (data not shown).

### 2.3. Pararama Hair Extract Induces the Production of Cytokines and Chemokines in Human Blood

**Figure 3:**
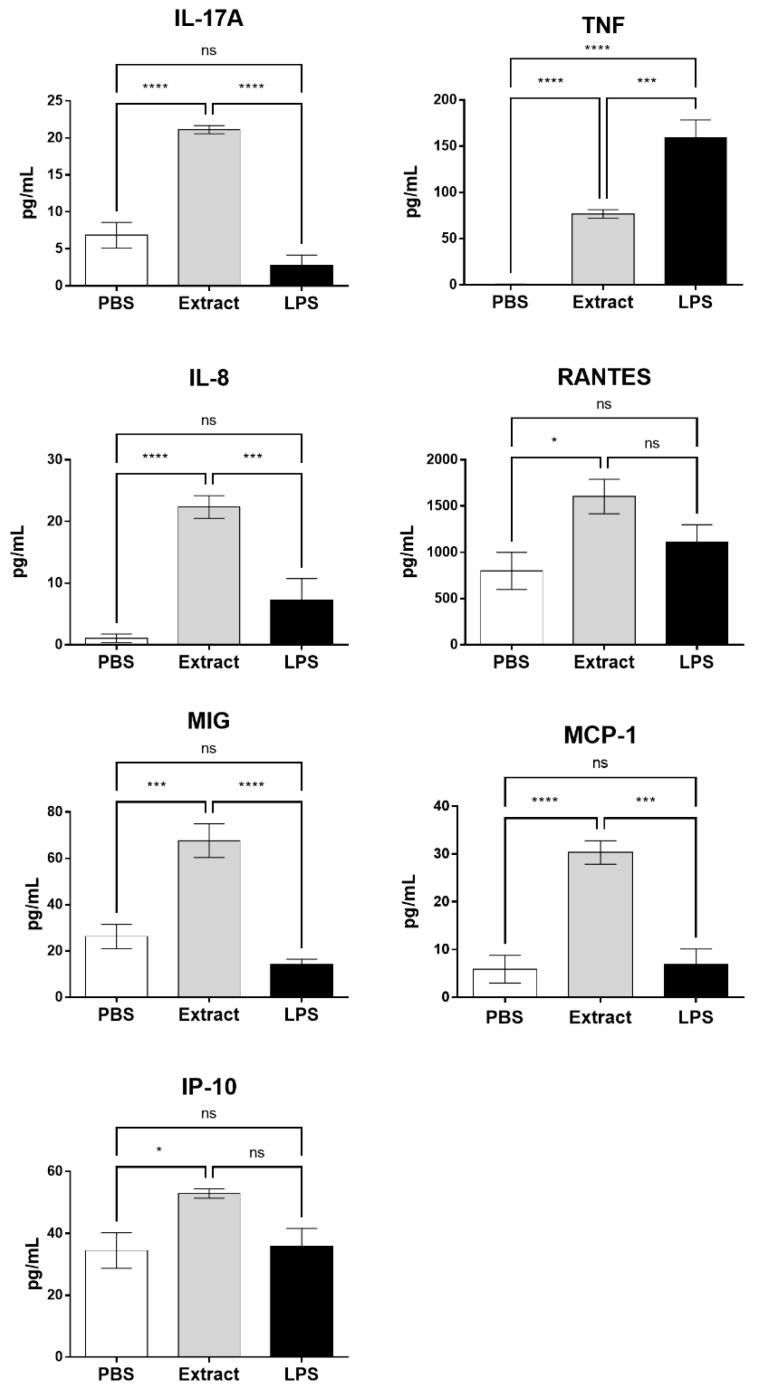
Cytokine and chemokine levels in whole blood stimulated with Pararama hair extract. Human blood samples anticoagulated with lepirudin (50 µg/mL) were incubated with Pararama hair extract (95 µg/mL), LPS (5 µg/mL), or PBS for 30 min at 37 °C. After plasma collection, the production of cytokines (IL-17A, TNF) and chemokines (IL-8, RANTES, MIG, MCP-1, and IP-10) was determined by flow cytometry. The results are presented as the mean ± SD of three independent experiments. * *p*: < 0.05. *** *p*: < 0.001. **** *p*: < 0.0001. ns: not significant.

Cytokine and chemokine production was evaluated in plasma after treatment of blood samples with Pararama hair extract, PBS, or LPS. Figure 3 shows that the extract induced a significant increase in the production of two cytokines that play an important role in the development of RA and OA, i.e., IL-17 and TNF-α, and was unable to induce the production of IL-2, IL-4, IL-6, IL-10, and IFN-γ (data not shown). LPS did not induce a significant increase in IL-17 production; however, LPS induced a TNF-α level that was significantly higher than the TNF-α level induced by the extract. The Pararama hair extract induced a significant increase in the production of the chemokines IL-8, RANTES, MIG, MCP-1, and IP-10 compared to the negative control. Regarding LPS, the difference was significant only for IL-8, MIG, and MCP-1. LPS, at the concentration used in the assay, did not induce a significant increase in any of the analyzed chemokines in relation to the negative control.

### 2.4. Compstatin Inhibits C3a, C5a, and TCC Generation Induced by Pararama Hair Extract in Human Blood

**Figure 4:**
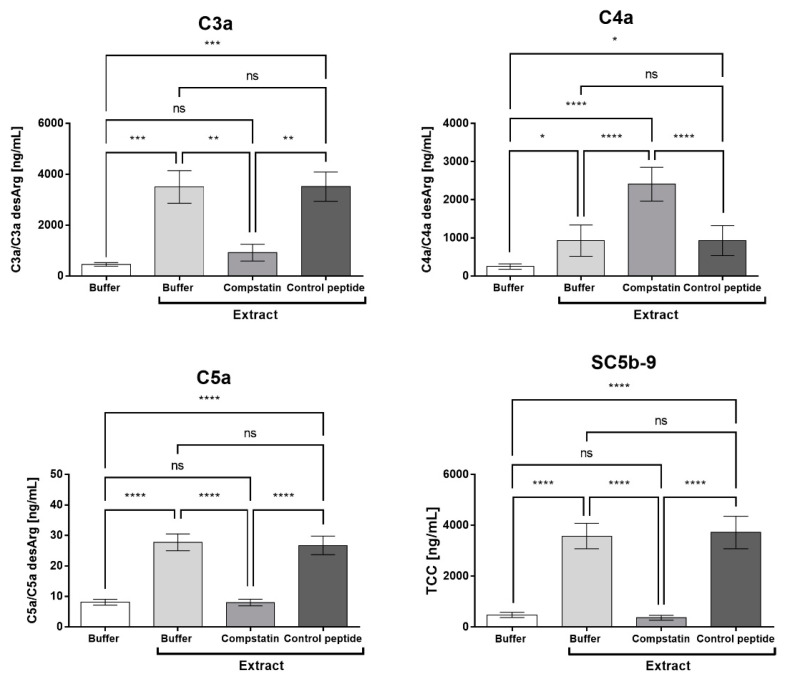
Production of anaphylatoxins and the terminal complement complex in whole blood stimulated with Pararama hair extract in the presence of compstatin. Human blood samples anticoagulated with lepirudin (50 µg/mL) were preincubated with PBS, compstatin (50 µM), or control peptide (50 µM) and then incubated with Pararama hair extract (95 µg/mL) or PBS for 30 min at 37 °C. After plasma collection, anaphylatoxin (C3a, C4a, and C5a) generation was assessed by flow cytometry, and SC5b-9 production was assessed by ELISA. The results are presented as the mean ± SD of three independent experiments. * *p*: < 0.05. ** *p*: < 0.01. *** *p*: < 0.001. **** *p*: < 0.0001. ns: not significant.

Figure 4 shows that compstatin was able to inhibit the generation of C3a, C5a, and the soluble terminal complement complex (SC5b-9) and that the control peptide did not interfere with this generation induced by the Pararama hair extract. However, compstatin was not able to control the production of C4a induced by the extract.

### 2.5. Compstatin Modulates Surface Cell Marker Expression Induced by Pararama Hair Extract in Granulocytes and Monocytes

**Figure 5:**
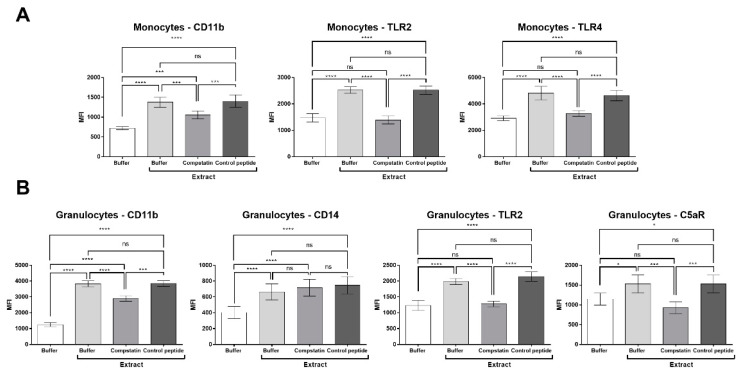
Expression of surface markers in monocytes and granulocytes stimulated with Pararama hair extract in the presence of compstatin. Human blood samples anticoagulated with lepirudin (50 µg/mL) were preincubated with PBS, compstatin (50 µM), or control peptide (50 µM) and then incubated with Pararama hair extract (95 µg/mL) or PBS for 30 min at 37 °C. After lysis of erythrocytes, cells were stained with anti-CD33 (monocytes), anti-CD66b (granulocytes), anti-CD11b, anti-CD14, anti-TLR2, anti-TLR4, and anti-C5aR. The expression of these markers was evaluated by flow cytometry, and the results are expressed as the median fluorescence intensity (MFI) after the acquisition of 20,000 events. The results are presented as the mean ± SD of three independent experiments. (**A**): Monocytes. (**B**): Granulocytes. * *p*: < 0.05. *** *p*: < 0.001. **** *p*: < 0.0001. ns: not significant.

Figure 5A shows that the inhibition of the complement system promoted a significant decrease in the expression of CD11b, TLR2, and TLR4 induced by Pararama hair extract in monocytes. In granulocytes, the presence of compstatin produced a significant decrease in the expression of CD11b, TLR2, and C5aR induced by Pararama hair extract (Figure 5B). The expression of CD14 was not modulated by the treatments, and the control peptide did not interfere with the tests performed, showing similar results with the human blood samples stimulated only with the hair extract of the *P. semirufa* caterpillar.

### 2.6. Compstatin Modulates IL-17, IL-8, RANTES, and MCP-1 Production Induced by Pararama Hair Extract in Human Blood

**Figure 6:**
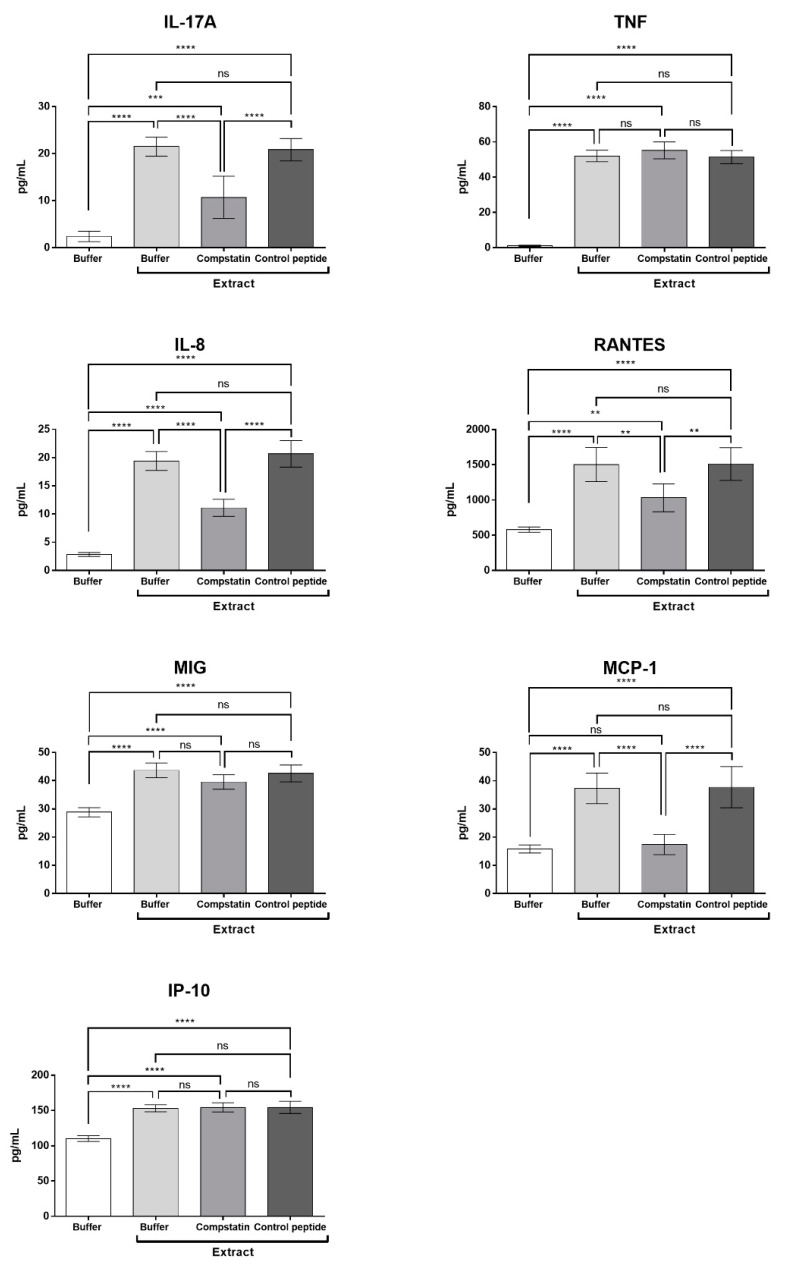
Cytokine and chemokine levels in whole blood stimulated with Pararama hair extract in the presence of compstatin. Human blood samples anticoagulated with lepirudin (50 µg/mL) were preincubated with PBS, compstatin (50 µM), or control peptide (50 µM) and then incubated with Pararama hair extract (95 µg/mL) or PBS for 30 min at 37 °C. After plasma collection, the production of cytokines and chemokines was determined by flow cytometry. The results are presented as the mean ± SD of three independent experiments. ** *p*: < 0.01. *** *p*: < 0.001. **** *p*: < 0.0001. ns: not significant.

To assess whether inhibition of the complement system with compstatin could interfere with the cytokine and chemokine production induced by the Pararama hair extract, human blood samples were preincubated with compstatin or control peptide and then treated with the extract or PBS. Figure 6 shows that in the presence of compstatin, there was a significant decrease in the production of IL-17A induced by Pararama hair extract; however, compstatin did not interfere with the production of TNF-α.

Inhibition of the complement system by compstatin promoted a significant decrease in the production of the chemokines IL-8, RANTES, and, mainly, MCP-1 that returned to basal levels. However, compstatin did not interfere with the production of MIG and IP-10 induced by the Pararama hair extract (Figure 6).

### 2.7. C5a is Involved in the Production of IL-8 and RANTES Induced by Pararama Hair Extract in Human Blood

**Figure 7:**
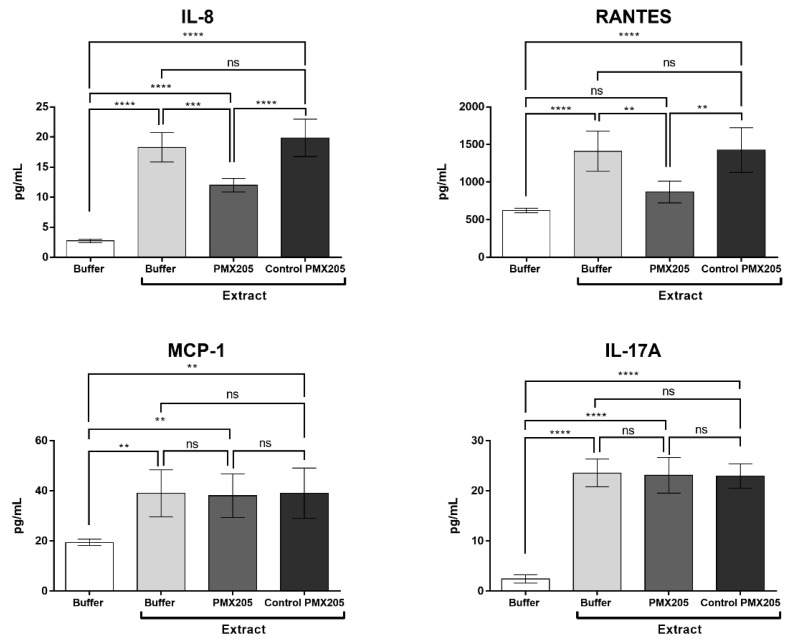
Cytokine and chemokine levels in whole blood stimulated with Pararama hair extract in the presence of PMX205. Human blood samples anticoagulated with lepirudin (50 µg/mL) were preincubated with PBS, PMX205 (10 µM), or PMX control vehicle and then incubated with Pararama hair extract (95 µg/mL) or PBS for 30 min at 37 °C. After plasma collection, the production of cytokines and chemokines was determined by flow cytometry. The results are presented as the mean ± SD of three independent experiments. ** *p*: < 0.01. *** *p*: < 0.001. **** *p*: < 0.0001. ns: not significant.

C5a induces or amplifies several innate immune responses and promotes most of its functions through interaction with C5a receptor 1 (C5aR1). To evaluate the role of C5a in cytokine and chemokine production, human blood samples were preincubated with the C5aR1 antagonist PMX205 and incubated with Pararama hair extract or PBS. Figure 7 shows that the inhibition of C5aR1 significantly decreased the production of the chemokines IL-8 and RANTES but did not interfere with the MCP-1 and IL-17A production induced by Pararama extract.

### 2.8. Pararama Hair Extract Induces IL-8 and MCP-1 Production in Eahy926 Human Endothelial Cells

**Figure 8:**
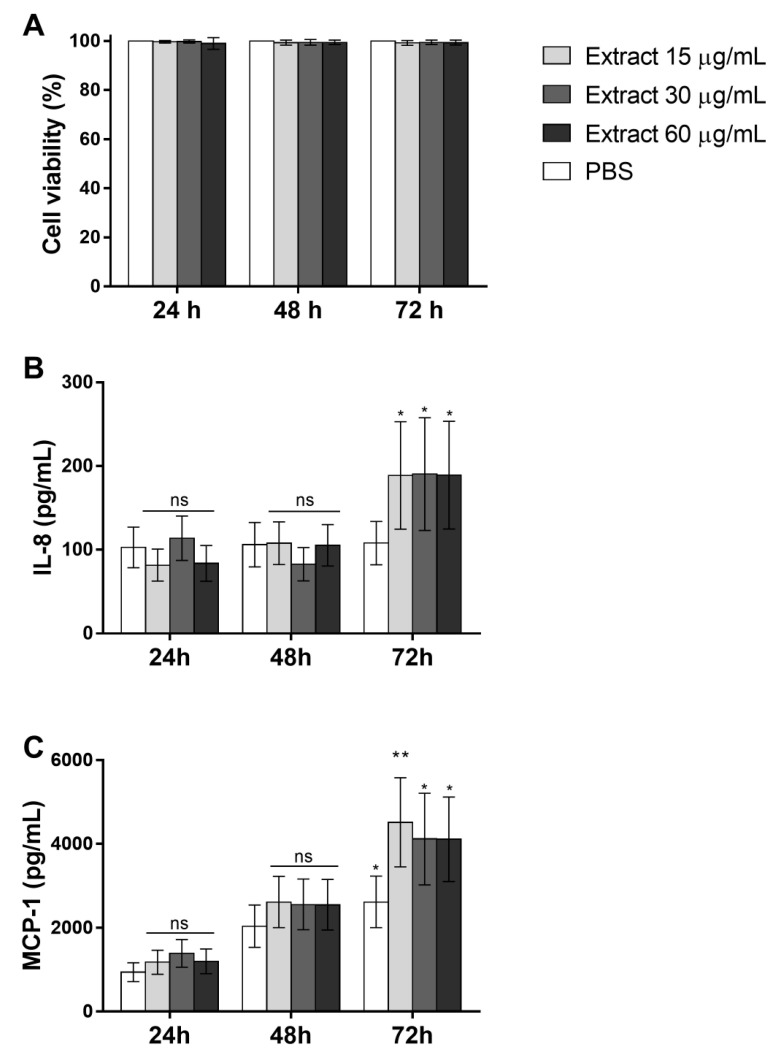
Cell viability and chemokine levels in cultures of EA.hy926 cells stimulated with Pararama hair extract. The endothelial cell line EA.hy926 (5 × 10^4^ cells/well) was treated with 15, 30, or 60 µg/mL extract or PBS for 24, 48, and 72 h in a CO_2_ incubator. After stimulation, the production of IL-8 and MCP-1 was determined in the supernatants of the cultures, and cell viability, expressed as a percentage, was evaluated by the MTT assay. The results are presented as the mean ± SD of three independent experiments. (**A**): Cell viability. (**B**): IL-8 levels. (**C**): MCP-1 levels. * *p* < 0.05; ** *p* < 0.01. ns: not significant.

To analyze whether the Pararama hair extract has a direct action on endothelial cells, EAhy926 cells were treated with 15, 30, and 60 μg/mL extract or PBS for 24, 48, and 72 h. After stimulation, cytokine and chemokine production in the supernatant of cultures and cell viability was assessed. The Pararama hair extract did not affect the viability of the cells at any of the times and concentrations tested (Figure 8A). (Figure 8B,C) shows that the extract induced the secretion of the IL-8 and MCP-1 chemokines at all concentrations used after 72 h of stimulation.

### 2.9. Complement Activation Products Induced by Pararama Hair Extract are Involved in IL-8 and MCP-1 Production by Endothelial Cells

To evaluate whether the complement activation products induced by the extract could stimulate endothelial cells to produce chemokines and cytokines, EAhy926 cells were incubated with plasma collected from human blood samples stimulated with PBS, extract, or extract + compstatin. Similar final concentrations (~1.9 µg) of the hair extract, present in the plasma samples, were used in the cultures of endothelial cells, and no secretion of the cytokines and chemokines was detected (data not shown).

**Figure 9:**
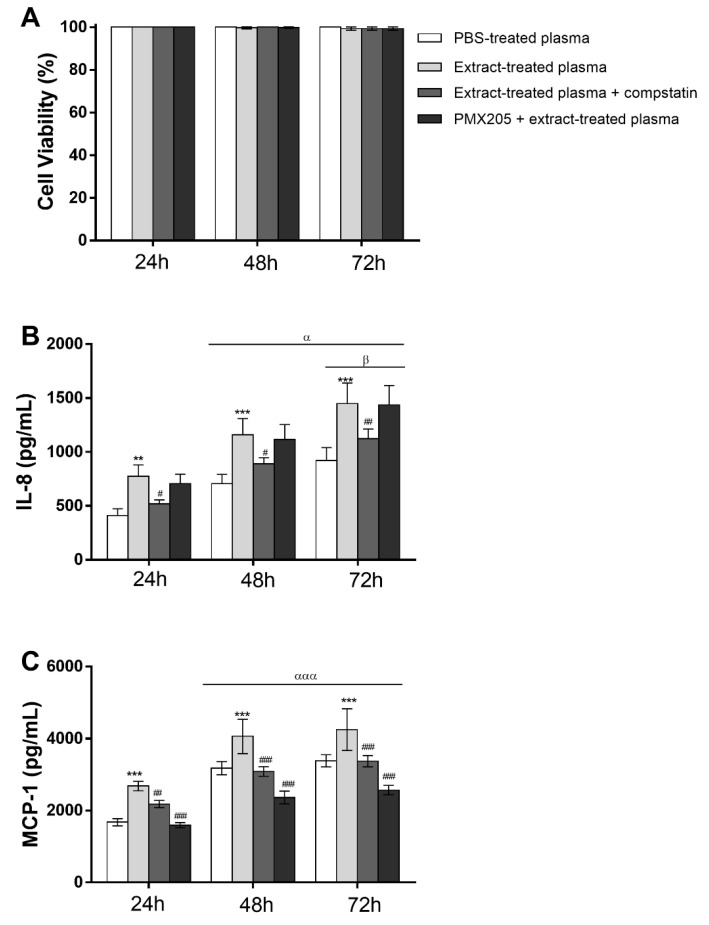
Cell viability and chemokine levels in cultures of EA.hy926 cells stimulated with Pararama hair extract-treated plasma. The endothelial cell line EA.hy926 (5 × 10^4^ cells/well) was treated with plasma (1:10 in culture medium) collected from blood samples stimulated with PBS (PBS-treated plasma), Pararama hair extract (extract-treated plasma) and Pararama hair extract in the presence of compstatin (extract-treated plasma and compstatin). After stimulation, the production of IL-8 and MCP-1 was determined in the culture supernatants by flow cytometry. To evaluate the role of C5a, Pararama hair extract-treated plasma in the presence of PMX205 was used in cultures (PMX205+ extract-treated plasma). The results are presented as the mean ± SD of three independent experiments. ** *p* < 0.01 compared to PBS-treated plasma. *** *p* < 0.001 compared to PBS-treated plasma. # *p* < 0.05 compared to Pararama hair extract-treated plasma. ## *p* < 0.01 compared to Pararama hair extract-treated plasma. ### *p* < 0.001 compared to Pararama hair extract-treated plasma. ^α^ *p* < 0.05 compared to 24 h. ^ααα^ *p* < 0.001 compared to 24 h. ^β^ *p* < 0.05 compared to 48 h.

The role of C5a in these assays was assessed using the C5aR1 antagonist PMX205 in the cell culture. The MTT assay was performed to assess whether the plasma stimulus was capable of compromising endothelial cell viability. The cells remained viable after all the stimuli and periods tested (Figure 9A). (Figure 9B,C) shows that Pararama hair extract-treated plasma, at all times analyzed, induced a significant increase in IL-8 and MCP-1 production compared to the control (PBS-treated plasma). Complement system inhibition with compstatin significantly decreased the chemokine production induced by Pararama hair extract-treated plasma. However, the incubation of endothelial cells with extract-treated plasma in the presence of PMX205 did not modulate the production of IL-8, indicating that C5a is not involved in the production of this chemokine in these cells. However, the use of the C5aR1 antagonist significantly decreased the production of MCP-1 in all periods of stimulation.

## 3. Discussion

Pararamosis is a neglected tropical disease affecting mainly rubber tappers living in the Amazonian region, and to date, no specific treatment has been developed for this disease.

To evaluate the inflammatory effect of the extract in a system where the interaction of cells and blood components occurs and that also allows determination of the involvement of the complement system in this effect, the ex vivo human whole blood model was used. This model allows pharmacological interventions to be carried out at specific points of the complement system cascade, such as the use of compstatin, a peptide that binds to C3, preventing its proteolytic cleavage by C3 convertase [39,40] and the complement C5a receptor 1 (C5aR1) antagonist PMX205 [41,42].

After treatment of human blood samples with the *P. semirufa* hair extract, there was a significant increase in the generation of anaphylatoxins (C3a, C4a, and C5a) and sTCC as well as the production of the cytokines TNF-α and IL-17 and the chemokines IL-8, RANTES, MIG, MCP-1, and IP-10.

In osteoarthritis (OA), the most common joint disease that can develop as a sequel of joint cartilage trauma [43] and evolve to cartilage and bone destruction, the role of complement was suggested 15 years ago [44] and still remains a promising field for investigation, since patients with OA had increased synovial fluid concentrations of C3a, which can be generated from all three pathways, compared to levels in healthy control subjects [19]. However, a detailed pathogenetic pathway triggered in the joint by complement activation and its crosstalk with other signaling routes contributing to OA remains poorly characterized.

Synovial fibroblasts and chondrocytes isolated from patients with OA, when stimulated in vitro with IL-17, have been shown to produce IL-8 and MCP-1 (chondrocytes) and IL-8 (fibroblasts), which suggests that IL-17 may contribute to cartilage degradation and synovial infiltration in OA [45]. Additionally, a positive correlation was shown between the serum level of IL-17 and primary knee osteoarthritis, which may reflect the severity of the disease [46]. The effects of IL-17 on chondrocytes and synovial fibroblasts derived from cartilage and synovium of patients with end-stage knee OA were evaluated by Mimpen and collaborators [47], who showed an increase in the gene expression of IL-6, CXCL1, CXCL2, CXCL3, CXCL3, MMP1, and MMP3. Tumor necrosis factor (TNF) may play an important role in the inflammatory responses that occur during osteoarthritis [48,49,50], triggering the production of IL-6 [51], IL-8 [52], and RANTES [53,54] by human articular chondrocytes [55]. In the present study, we observed that *P. semirufa* venom induced the production of IL-17 and TNF, and complement inhibition at the C3 level decreased IL-17 production.

Anticomplement therapy in models of disease has been widely explored in recent decades, such as the use of cobra venom factor (CVF) to inhibit disease in animal models [56], and increasing efforts are being made to produce selective complement inhibition by employing monoclonal antibodies against complement components or by administering specific inhibitors [57].

The C3 blocker compstatin, administered intravitreally in nonhuman primates, has shown a reversal of drusen formation by suppression of complement activation in age-related macular degeneration (AMD) [58]. The standard procedure for patients with paroxysmal nocturnal hemoglobinuria (PNH) with significant clinical symptoms is the humanized monoclonal antibody eculizumab (Soliris), which binds to C5 and blocks the terminal complement pathway and was approved in 2007 [59,60]. Other anti-C5 antibodies have also been developed and are currently in clinical trials, such as tesidolumab, also known as LFG316, which is undergoing testing for AMD (NCT01527500), PNH (NCT02534909) and non-infectious intermediate, posterior or panuveitis (NIIPPU) (NCT01526889); ravulizumab, also called ALXN1210, is in phase 3 clinical trials for PNH (NCT03056040), substituting in the treatment for eculizumab, with results not inferior to eculizumab [61].

Pegcetacoplan (APL-2 or EMPAVELI), a long half-life form derivative from compstatin, is the first C3-targeted PNH therapy to be approved (in May 2021) in the USA [62,63,64], and its regulatory assessment is currently underway in the EU and Australia [63]. Another compstatin derivative, AMY-101 (Cp40), is currently in phase II clinical trials in adults with gingivitis (NCT03694444) [65,66] and for the management of patients with acute respiratory distress syndrome (ARDS) caused by SARS-CoV-2 infection (NCT04395456) [67,68] and has demonstrated good safety and tolerability in human volunteers in a phase I study (NCT03316521).

The C5aR1 blocker, the cyclic hexapeptide PMX53, modeled on the C-terminus of C5a, showed high specificity and affinity for C5aR1 in many species. Several phase I and IIa clinical trials with PMX53 were initiated for diseases such as rheumatoid arthritis, osteoarthritis, psoriasis, and AMD. The molecule proved to be safe but with low oral bioavailability resulting in poor efficacy. However, a modified version of PMX53, PMX205, showed improvements in these parameters and improved brain penetrance and thus may be more suitable for future clinical trials [69,70,71]. In rodent models, PMX205 has shown beneficial effects in amyotrophic lateral sclerosis [72,73], striatal neurodegeneration [74], spinal cord injury [75,76], reduction of memory loss in mice with Alzheimer’s disease [77,78], and in *Naja annulifera* snake envenomation, demonstrating the importance of the C5a-C5aR1 axis in the immunopathology caused by this venom [79].

In our study, the inhibition of C3 by compstatin significantly decreased IL-17, IL-8, RANTES, and MCP-1 production, indicating a possible involvement of the complement system in the production of these molecules. However, the use of the C5aR1 antagonist PMX205 promoted a reduction in the production of IL-8 and RANTES. Since PMX205 did not alter the production of IL-17 and MCP-1, it is possible to consider that these cytokines were stimulated by the extract-induced C5a anaphylatoxin. With the use of compstatin, maintenance of the generation of anaphylatoxin C4a was observed, likely because compstatin binds to the C3 component, preventing its cleavage, but does not interfere with the action of C1 and MASPs, which are responsible for the cleavage of the C4 component, in C4a and C4b. This increase can also occur by direct cleavage of C4 by metalloproteinases present in Pararama hair extract, as already demonstrated by Villas Boas et al. [10]. As the production of TNF, MIG, and IP-10 was not affected by compstatin or PMX205, we can conclude that their release was independent of C3a and C5a. In our assay, the release of these mediators could therefore be induced by C4a or by the direct action of Pararama extract on leukocytes or through the activation of other plasma compounds. This axis remains to be further investigated.

The use of compstatin in our model also decreased CD11b, TLR2, and C5aR1 expression induced by *P. semirufa* caterpillar hair extract in granulocytes, and CD11b, TLR4, and TLR2 in monocytes suggest that complement system activation and the interaction of its products with some receptors may contribute to the progression of pararamosis.

The vascular endothelium contributes to and is affected by inflammatory processes. Exposure of the endothelium to mechanical, chemical, or immunological stimuli induces a proinflammatory state with progressive loss of fundamental physiological functions. The activated endothelium is characterized by increased expression of adhesion molecules and cytokine/chemokine secretion, resulting in increased permeability to leukocyte migration. A variety of stimuli are able to activate endothelial cells, such as LPS, IL-1, TNF-α, and lipid derivatives [80,81].

The endothelium can also be activated by products generated by complement system activation [82]. Endothelial cells exposed to TCC may undergo lysis but often release and express molecules involved in important biological functions [83]. TCC deposition in endothelial cells promoted NF-κB activation and secretion of IL-8 and MCP-1 [84]. C5a anaphylatoxin is a powerful stimulus for the activation of endothelial cells, inducing increased expression of P-selectin, von Willebrand factor secretion [85], superoxide generation [86], and the production of IL-8 and MCP-1 [87].

When we incubated Ea.hy926 cells with extract-treated plasma, there was an increase in IL-8 and MCP-1 production, and complement system inhibition at the C3 level using compstatin in the whole blood assay was able to decrease the production of these chemokines. The incubation of these cells with the extract-treated plasma in the presence of PMX205 did not interfere with the production of IL-8, indicating that C5a is not involved in the production of this chemokine in our model, suggesting that this occurs due to high levels of C3a or TCC present in the extract-treated plasma. However, the use of this C5aR1 antagonist decreased the production of MCP-1 in endothelial cells, indicating a strong role of extract-induced C5a anaphylatoxin in the activation of endothelial cells.

In conclusion, the data obtained here show that the human whole blood model, together with the EA.hy926 endothelial cell activation model, is useful for studying the role of complement system activation in the inflammatory process caused by contact with *P. semirufa* caterpillar hairs. Furthermore, the data suggest that inhibition of the complement system at the level of C3 or C5aR1 may be useful in relieving inflammation and pararamosis progression.

## 4. Materials and Methods

### 4.1. Premolis Semirufa Hair Extract

Caterpillars from *P. semirufa* were collected in São Francisco, Pará, Brazil (capture and maintenance licenses from Brazilian Institute of Environment and Renewable Natural Resources (IBAMA), Brazil, number 45166–6). The caterpillar hairs were cut and transferred to tubes containing phosphate-buffered saline (PBS) buffer (8.1 mM Na_2_HPO_4_; 1.5 mM KH_2_PO_4_; 137 mM NaCl; 2.7 mM KCl; pH 7.4) and frozen at −80 °C until use. To prepare the extract, samples were macerated with a glass rod, and the insoluble material was removed by centrifugation at 560× *g* for 20 min at 4 °C. The supernatant was filtered through a 0.22 μm membrane (Whatman-GE Healthcare, Chicago, IL, USA), aliquoted, and stored at −80 °C (Access to Genetic Heritage n° AEA2993). The protein concentration was determined using the bicinchoninic acid (BCA) Protein Assay kit (Pierce Biotechnology, Rockford, IL, USA) according to the manufacturer’s recommendations.

The presence of endotoxin contamination in the Pararama extract was determined using PYROGENT™ Plus Gel Clot LAL Assays (Lonza, MD, USA) by the Microbial Control Unit of Butantan Institute (São Paulo, SP, Brazil). Endotoxin contamination was below the detection level (0.125 UE/mL), indicating that the results obtained in this study were due to the Pararama extract effect.

### 4.2. Human Whole Blood Model

Blood samples were collected from healthy consenting donors at the Ambulatory of the Butantan Institute (Human Research Ethics Committee from the University of São Paulo, São Paulo, Brazil, certificate number 1452/18). Blood was collected by venipuncture into tubes containing 50 μg/mL lepirudin (Refludan, Celgene, Munich, Germany), the recombinant form of hirudin with an anticoagulant activity that does not interfere with complement system activation [88].

### 4.3. Blood Sample Treatment

After collection, human blood samples (72% of the total volume, *v/v*) were incubated with a previously validated concentration of Pararama hair extract that activates the complement system in normal human serum (95 μg/mL–Villas Boas et al. [10]), LPS (lipopolysaccharide from *Escherichia coli* strain O111:B4, Sigma-Aldrich, St. Louis, MO, USA; 5 μg/mL) or PBS (28% of total volume, *v/v*) for 30 min in a water bath at 37 °C under agitation. Then, the tubes were centrifuged at 404× *g* at 4 °C for 10 min, and plasma samples were recovered. Plasma samples were aliquoted and stored at −80 °C in the presence of ethylenediaminetetraacetic acid (EDTA) (10 mM) for further analysis. The pellet was resuspended in red cell lysis buffer (Lysing Solution-BD Biosciences, Franklin Lakes, NJ, USA) and incubated for 10 min at room temperature. After incubation, the material was centrifuged at 720× *g* at 4 °C for 10 minutes and resuspended in PBS-paraformaldehyde 0.5% for analysis of the expression of the cell surface markers by flow cytometry in BD FACSCanto II (BD Biosciences, San Jose, CA, USA).

### 4.4. Complement System Inhibition

For complement inhibition, we used Compstatin, a 13 amino acid cyclic peptide (ICVVQDWGHHRCT) isolated from a random peptide library that binds to C3 and inhibits its proteolytic cleavage by the C3 convertase. In parallel to the use of compstatin, we used a control peptide (IAVVQDWGHHRAT), which completely lacks complement-inhibitory activity (Sahu et al., 1996) (Tocris Bioscience, Bristol, UK). We also used PMX205, a cyclic peptide (hydrocinnamate-[Orn-Pro-dCha-Trp-Arg]) antagonist of C5aR1 [41].

Blood samples (72% of total volume, *v/v*) were incubated with PBS, compstatin (50 μM), or the control peptide (50 μM) for 5 min at room temperature (14% of the total volume, *v/v*). Alternatively, blood samples (72% of the total volume, *v/v*) were incubated with PBS, PMX205 (10 μM), or PMX205 dilution vehicle (5% glucose–Sigma-Aldrich, Missouri, USA) for 5 min at room temperature (14% of the total volume, *v/v*). In sequence, PBS or Pararama hair extract (95 μg/mL) (14% of the total volume, *v/v*) was added to the tubes. Samples were incubated at 37 °C in a water bath under agitation for 30 min. Then, the tubes were centrifuged at 404× *g* at 4 °C for 10 min, and the plasma samples were collected. The plasma samples were aliquoted and stored at −80 °C in the presence of EDTA (10 mM) for further analysis. The pellet was resuspended in red cell lysis buffer and incubated for 10 min at room temperature. After incubation, the material was centrifuged at 720× *g* at 4 °C for 10 min and resuspended in PBS-paraformaldehyde 0.5% for analysis of cell marker expression.

### 4.5. Detection of the Soluble C5b-9 Complex, Anaphylatoxins, Cytokines, and Chemokines in Plasma Samples

The generation of the soluble C5b-9 complex (sTCC) in plasma samples collected from human whole blood assays was evaluated by enzyme-linked immunoassay (ELISA) using the MicroVue SC5b-9 Plus EIA kit (Quidel Corporation, San Diego, CA, USA). Plasma levels of C3a/C3a-desArg, C4a/C4a-desArg, and C5a/C5a-desArg were determined using the BD Cytometric Bead Array (CBA) Human Anaphylatoxin Kit (BD Biosciences, San Jose, CA, USA). The measurements of cytokines (IL-2, IL-4, IL-6, IL-10, IL-17A, IFN-γ, and TNF) and chemokines (CXCL8/IL-8, CCL5/RANTES, CXCL9/MIG, CCL2/MCP-1 and CXCL10/IP-10) were performed using a BD cytometric bead array (CBA) Th1/Th2/Th17 Human cytokine kit (BD Biosciences) and CBA Human Chemokine Kit (BD Biosciences), respectively. All assays were performed according to the manufacturers’ instructions.

### 4.6. Analysis of Surface Marker Expression in Granulocytes and Monocytes

After lysing erythrocytes and fixing leukocytes with paraformaldehyde, cell populations were selected based on their forward- and side-scatter features and using mouse antihuman monoclonal antibodies: anti-CD66b labeled with Alexa Fluor 647 (A647) (clone G10F5, BD Biosciences) for granulocytes and anti-CD33 labeled with allophycocyanin (APC) (clone WM53, BD Biosciences) for monocytes.

The expression of C3aR, C5aR1, CD11b, CD14, TLR2, and TLR4 on the surface of these cells was analyzed using specific mouse antihuman monoclonal antibodies or isotype controls labeled with R-phycoerythrin (PE) or fluorescein isothiocyanate (FITC): anti-C3aR PE (clone hC3aRZ8, BD Biosciences); anti-C5aR1 FITC (clone 8D6, Santa Cruz Biotechnology, Dallas, TX, USA); anti-CD11b PE (clone ICRF44, Invitrogen, Thermo Fisher Scientific, Carlsbad, CA, USA); anti-CD14 FITC (clone 61D3, eBioscience, San Diego, CA, USA); anti-TLR2 PE (clone 11G7, BD Biosciences); anti-TLR4 PE (clone HTA125, eBioscience, CA, USA); mouse IgG2bκ PE isotype control (BD Biosciences); mouse IgG2a FITC isotype control (Dako, CPH, Glostrup, Hovedstaden, Denmark) and mouse IgG1κ PE isotype control (Dako). The samples were incubated with the antibodies for 30 min in the dark at room temperature and washed with flow cytometry staining buffer (FACS) (PBS containing 1% bovine serum albumin (BSA) for analysis in a FACSCanto II flow cytometer (BD Biosciences) using BD FACSDiva software, version 4.1 (BD Biosciences). The expression of cell surface markers was represented as the median fluorescence intensity (MFI) of stained cells after subtraction of the respective isotype control.

### 4.7. Direct and Indirect Action of Pararama Hair Extract on Endothelial Cells

The vascular endothelial cell line EAhy926 was cultured in Dulbecco’s modified Eagle’s medium (DMEM) (Gibco, Invitrogen Corp., Carlsbad, CA, USA) containing 10% (*v/v*) heat-inactivated fetal bovine serum (FBS; Cultilab, SP, Brazil) and 1% penicillin–streptomycin (Gibco, Invitrogen Corp., Carlsbad, CA, USA) at 37 °C and 5% CO_2_.

The cells were subcultured in 96-well plates (5 × 10^4^ cells/well) and maintained overnight in DMEM without FBS, followed by incubation with increasing concentrations of Pararama hair extract (15, 30, and 60 µg/mL), culture medium containing 10% plasma collected from whole blood samples treated with Pararama hair extract (extract-treated plasma), Pararama hair extract + compstatin (extract-treated plasma and compstatin), or PBS (PBS-treated plasma), for 24, 48 and 72 h at 37 °C and 5% CO_2_. To evaluate the role of C5a in cytokine/chemokine production by endothelial cells, we added the C5aR1 antagonist PMX205 in the presence of a culture medium containing 10% fresh plasma from whole blood samples treated with Pararama hair extract (PMX205+ extract-treated plasma) to the wells. After incubation, the supernatants were collected for the measurement of cytokines and chemokines, and cell viability was assessed by the MTT method [89].

### 4.8. Endothelial Cell Viability Analysis by MTT Assay

After supernatant collection, cells were incubated with MTT (0.5 mg/mL in 100 µL DMEM) for 4 h at 37 °C and 5% CO2. Then, the MTT solution was removed, and 100 µL of dimethyl sulfoxide (DMSO) was added to each well. The absorbance was measured in a spectrophotometer (Multiskan-EX, Labsystems, VANTAA, Uusimaa, Finland) at 540 and 620 nm. The relative cell viability was calculated as follows:

$$\mathrm{Cell}\;\mathrm{viability}\;(\%)=\frac{\left[\mathrm{Abs}\;540\;\mathrm{nm}–\mathrm{Abs}\;620\;\mathrm{nm}\right]\;\left(\mathrm{sample}\right)\times 100}{\left[\mathrm{Abs}\;540\;\mathrm{nm}–\mathrm{Abs}\;620\;\mathrm{nm}\right]\;\left(\mathrm{control}\right)}$$

### 4.9. Statistics

Data were analyzed by one-way or two-way analysis of variance (ANOVA) followed by Bonferroni’s post-test using GraphPad Prism software v.9.2 (GraphPad Software, San Diego, CA, USA). A *p*-value of <0.05 was considered significant.

## Data Availability

Not applicable.
